# Functional Language in Children from a Public Cochlear Implant Program in a Developing Country

**DOI:** 10.1055/s-0044-1785205

**Published:** 2024-05-25

**Authors:** Alice Lang Silva, Anderson Claudio Roberto, Maithe Antonello Ramos, Debora Milene Ferreira Alves, Isadora Martins Silva Stumpf, Laura Prolla Lacroix, Letícia Petersen Schmidt Rosito

**Affiliations:** 1 School of Medicine, Universidade Federal do Rio Grande do Sul, Porto Alegre, RS, Brazil; 2 Division of Otorhinolaryngology and Head and Neck Surgery, Hospital de Clínicas de Porto Alegre, Universidade Federal do Rio Grande do Sul, Porto Alegre, RS, Brazil

**Keywords:** prelingual deafness, cochlear implants, language development, sign language

## Abstract

**Introduction** The World Health Organization (WHO) estimates that ∼ 32 million children worldwide are affected by hearing loss (HL). Cochlear implant is the first-line treatment for severe to profound sensorineural HL. It is considered one of the most successful prostheses developed to date.

**Objective** To evaluate the oral language development of pediatric patients with prelingual deafness implanted in a reference hospital for the treatment of HL in southern Brazil.

**Methods** We conducted a retrospective cohort study with a review of medical records of patients undergoing cochlear implant surgery between January 2009 and December 2018. Language development was assessed by reviewing consultations with speech therapy professionals from the cochlear implant group.

**Results** A total of 152 children were included in the study. The mean age at cochlear implant surgery was of 41 months (standard deviation [SD]: ± 15). The patients were divided into six groups considering the type of language most used in their daily lives. We found that 36% of children use oral language as their primary form of communication. In a subanalysis, we observed that patients with developed or developing oral language had undergone cochlear implant surgery earlier than patients using Brazilian Sign Language (Língua Brasileira de Sinais, LIBRAS, in Portuguese) or those without developed language.

**Conclusion** The cochlear implant is a state-of-the-art technology that enables the re-establishment of the sense of hearing and the development of oral language. However, language development is a complex process known to present a critical period to properly occur. We still see many patients receiving late diagnosis and treatment, which implies a delay and, often, the impossibility of developing oral communication.

**Level of Evidence** Level 3 (cohort study).

## Introduction

The World Health Organization (WHO) estimates that ∼ 32 million children worldwide are affected by hearing loss (HL). 1 When left untreated during childhood, it can lead to impaired language acquisition throughout life, with negative repercussions on interpersonal communication, cognition, school performance, and psychosocial well-being. 2 3

Cochlear implant (CI) is the first-line treatment for severe to profound sensorineural HL. It is considered one of the most successful prostheses developed to date, and it has revolutionized the natural history of profound deafness. 1 4 It works by transforming the acoustic signal into an electrical stimulus, directly activating the auditory nerve fibers when the cochlea is not functional. Therefore, children with prelingual deafness are given the opportunity to hear and, consequently, to develop oral language.

Nevertheless, individual progress can vary widely, reflecting the conditions of health systems and the uniqueness of each child. This variability in response to the use of CI is the subject of research around the world. One of the ways to determine the prognostic factors associated with oral language development in a given population is to evaluate the characteristics of groups who present good and poor performances.

The number of studies dedicated to evaluating CI outcomes in terms of language acquisition in developing countries is limited. In Brazil, one of the many reasons for this is the fact that the population of prelingually deaf children implanted in the country is heterogeneous. The differences range from age at implantation, multiple etiologies, and socioeconomic status, leading to unequal access to postoperative rehabilitation. 5

The first CI in Brazil dates to 1977, the second to be performed outside the United States. 6 The CI program in the Brazilian public health system (Sistema Único de Saúde, SUS, in Portuguese) began in the 1990s, and now has more than 40 centers registered to perform this surgery around the country. 7 National research about oral language development in prelingual patients has been published, but they usually evaluate specific subgroups or describe only a small sample of patients. 8 9 10

The present study describes functional oral language development after CI surgery for prelingual deafness among pediatric patients from a public health system program. In addition, we relate the loss to follow-up rate, the mean age at CI, the main etiologies, and the association of these factors with speech development.

## Methods

Pediatric patients who underwent CI surgery at our hospital from January 2009 to December 2018 were selected for a retrospective cross-sectional study. Most children had been implanted before simultaneous bilateral cochlear implant surgery was the standard treatment for children in the public health system, which started in 2018. The inclusion criteria were as follows:

- Profound bilateral HL;
- Cochlear implant performed through the SUS;
- Age ≤ 6 years at the time of CI surgery;
- Congenital HL or HL acquired in the neonatal period;
- Having at least 12 months of postoperative follow-up.
  The appraisal of oral language development was performed through medical chart review. We examined all speech therapy consultations of each patient to evaluate the primary form of communication used by the child. According to that review, the patients were classified into six functional stages of language development conceived by the authors:
  1. Established oral language (EOL): patients who use only oral language for communication and showed speech comprehensibility;
  2. Developing oral language (DOL): patients who had been implanted more recently and were considered to present adequate oral language development considering the evolution time;
  3. Mixed: patients who use oral language but also attended sign language schools and could be understood in both languages.
  4. Brazilian sign language (Língua Brasileira de Sinais, LIBRAS, in Portuguese): patients who did not develop speech and ended up choosing or being referred to learn sign language;
  5. Undetermined: patients who dropped out of the CI program within the first two years after undergoing surgery;
  6. No language: patients who were still being followed but had not yet developed consistent oral or sign language.

Regarding the use of CIs, the medical records were reviewed to identify if the patient's caregiver consistently reported the correct use of the CI during the day or if the patient was no longer using their device. In the latter case, we subdivided the group into those who were no longer using the CI as an option and those who were not using it because of maintenance/misusage issues.

In addition, we evaluated the proportion of patients lost to follow-up, the mean age at CI surgery, and its association with language outcomes. For the statistical analysis, we used the Kruskal-Wallis statistical test within Statistical Package for the Social Sciences (SPSS, SPSS Inc., Chicago, IL, United States) software, version 18.0. The present study was part of a project approved by the Ethics in Research Committee of our hospital (under protocol number 15–0445).

## Results

From January 2009 to December 2018, 201 pediatric patients underwent CI surgery at our hospital and were included in the study. After reviewing the medical charts, we found that 24.3% of the patients included had missed the medical, audiologist's, and speech therapist's consultations for more than one year, so they were considered lost to follow-up. Those patients were not analyzed in terms of language development.

A total of 152 children met the inclusion criteria and had their medical charts reviewed. Most of the sample had received only one CI, but 9% had undergone a sequential bilateral CI surgery. Patients who had the second surgery performed at that time did it either because the family got health insurance after the first procedure or by legal means. Other characteristics of the sample can be found in Table 1 .

**Table 1:** Characteristics of the study sample

Characteristic	
**Gender: n (%)**	
Female	77 (50.6)
Male	75 (50.4)
**Stimulation modality: n (%)**	
Bilateral	14 (9.2)
Unilateral	138 (90.8)
**Age at first CI (in months): mean(± SD); range**	
General	41(± 15.7); 14–71
**Etiology: n (%)**	
Inner ear malformation	3 (1.9)
Congenital infection	4 (2.6)
Auditory neuropathy	5 (3.2)
CNS malformation	5 (3.2)
Genetic (non-syndromic)	6 (3.9)
Genetic (syndromic)	8 (5.2)
Meningitis	12 (7.9)
Neonatal conditions	28 (18.5)
Unknown	82 (53.6)

Abbreviations: CI, cochlear implant; CNS, central nervous system; SD, standard deviation.

**Graph 1:**
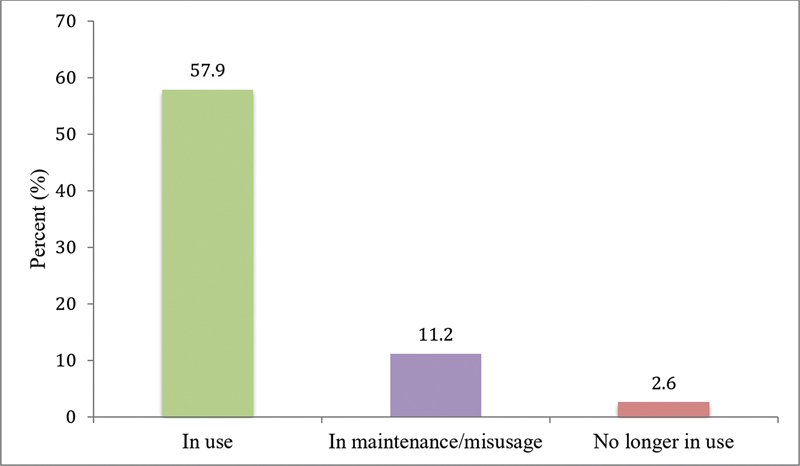
Proportion of cochlear implant use.

In regard to CI use, the number of patients who were consistently using the CI, those who were no longer using the CI as an option, and those who were not using it because of maintenance/misusage issues can be found in Graph 1 .

**Graph 2:**
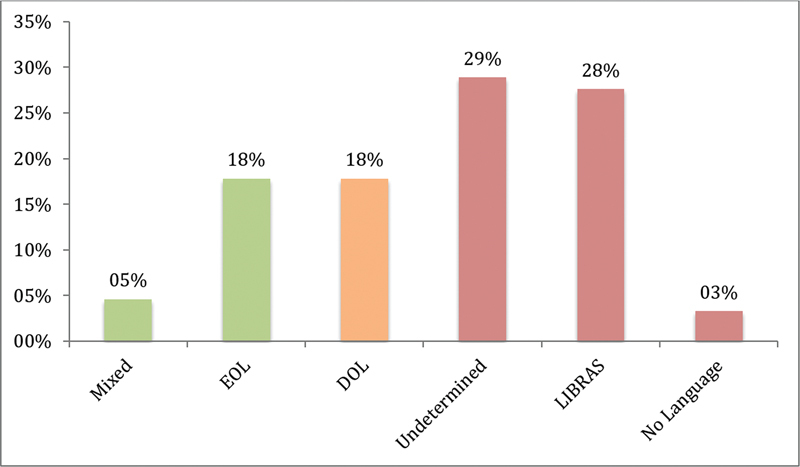
Percentage of patients in each group regarding language development. Mixed (use of oral and sign language); EOL (established oral language); DOL (developing oral language); LIBRAS (Língua Brasileira de Sinais – Brazilian Sign Language).

Regarding the aforementioned six functional stages of language development, the results from this group of implanted children are depicted in Graph 2 .

**Graph 3:**
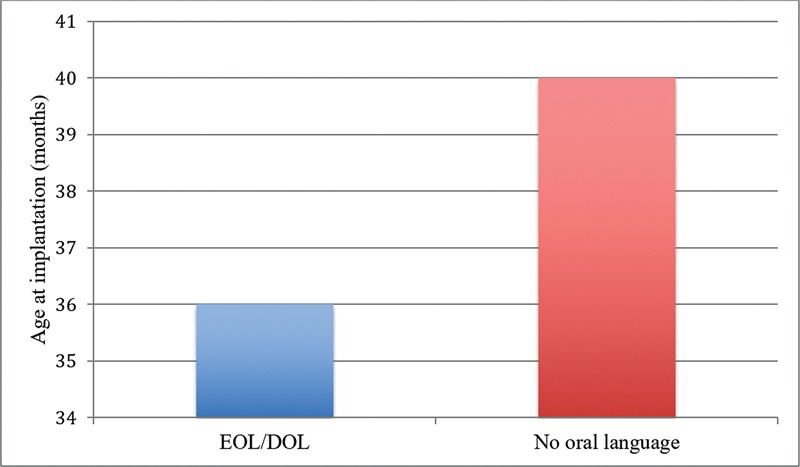
Mean age at which cochlear implant was performed in patients in the EOL/DOL groups versus patients in the LIBRAS or without any language groups.

When comparing the age at the time of CI surgery, we observed that children with established or developing oral language had been implanted earlier than children using LIBRAS or those without language, with a statistically significant difference ( *p* = 0.02), as shown in Graph 3 . Because of the large number of patients with an unknown etiology and very few patients in other etiology groups, the subgroup analysis in terms of language performance was not conducted.

## Discussion

The results of the present study show a significant challenge faced by our public CI program: the low proportion of prelingually deaf children who develop functional oral language after CI surgery. We acknowledge that this was a retrospective study that staged language development through a non-standardized classification and that it was not designed to explain the results. Still, it provides a starting point for many hypotheses to be explored.

First, a brief explanation about the choice to classify language by *functional* stages: at the beginning of our CI program, different tests were being used to assess language development by distinct speech therapists. When the present study was conceived, it was first thought of as a way to understand our results as assistance feedback. So, as a multidisciplinary team that involves doctors, audiologists, speech therapists, social service workers, and psychologists, we believed that a *functional* classification (that divided patients according to how much they used oral language in their daily lives) enabled the entire team to understand better how patients developed throughout the years. In that sense, even though it was not a standardized test that can be used worldwide to compare language development results, we believe that it served as a functional classification that enables health staff as well as the general public to understand how these patients can communicate with other people in their daily lives, which is the main objective of this surgery. Therefore, it may be considered a valuable tool for other CI programs in developing countries, which may find it difficult to analyze their results retrospectively.

An alarming finding was the unexpected proportion of patients who dropped out of the CI program. It must be stressed that regular appointments, especially in the first years after surgery, are considered critical, and parents are informed of that importance before surgery. They are given the opportunity to re-schedule appointments in case of any setbacks, and that is why patients who had missed consultations in the CI program for one year or more were considered lost to follow-up. Perhaps, those were the patients who had the poorest results in terms of general development, but a retrospective chart review would be a superficial method of analyzing that assumption. An active search for these patients with the help of the social service team is necessary and was one of the points raised with the multidisciplinary team, as it is an important finding to be explored.

Another critical data found was the advanced age at which CI surgeries were performed in our sample. Even though many studies in the international literature mention countless variables to explain good and poor results regarding oral language development, early age at implantation seems to be a consensus in terms of importance to achieve a good outcome. 11 12 13 14 Groups worldwide even discuss the differences between patients who undergo surgery before 12 months of age and those who undergo it later. 11 12 15 16 Our finding that children with established or developing oral language had been implanted earlier than children without oral language development is another result that points to the importance of this variable in our setting as well.

Unfortunately, performing CI before 24 months age in Brazil is still a challenging goal to be reached, especially for children who depend on the SUS. We know that the sooner the HL diagnosis is established, the greater are the chances of early treatment with a CI. However, in Brazil, one of the main problems related to early diagnosis is universal neonatal hearing screening (UNHS), which is not universal in practice, being performed in 37.2% of live births in the country according to a national survey. 17 Even though Brazil has an uneven distribution, with the lowest rates registered in the North and Northeast regions and better rates in the Southern states (in our state, Rio Grande do Sul, there are regions with coverage greater than 95%), we can infer from our results that flaws in UNHS are not the only problem leading to our poor results.

In a previous study performed by our group, 18 we found that the mean age at the first consultation for children referred to our specialized pediatric HL outpatient clinic was of 1.4 years. Those who had undergone UNHS were younger than those who had not. Nevertheless, the children who had passed the UNHS but were later diagnosed with HL reached the first appointment with a specialist and started treatment older than those who had failed. 18 This finding leads to two other conclusions about the results of the present study: 1) failing the UNHS should impact the early referral for specialized assistance and it is not achieving its goal; and 2) passing the UNHS should not reduce the attention of the network of children's caregivers regarding language development milestones, given the risk of progressive HL or even the possibility of auditory neuropathy, especially in children with risk factors.

The interpretation of the results obtained in the hearing screening and the importance of monitoring language milestones are not always straightforward for all health professionals who care for young children, not to mention for parents who have a low level of schooling. Recommendations from the 2007 and 2019 guidelines for early hearing detection and intervention programs are clear about the necessity of careful interpretation of results and surveillance of communication development. 19 20 While these documents are widely discussed among professionals from related fields (mainly audiologists and otolaryngologists), many of the children referred to our program had been previously evaluated only by primary care physicians. Despite not having precise data about the reasons for referrals for this sample of patients, it is our impression, as a team, that the first referrals to investigate HL only occur after the child's first year of life, when no language milestones have been achieved.

Other possible reasons that could explain these discouraging results could be drawn out from similar research. A study conducted in Canada 21 with 118 children revealed that late implantation (more than 12 months after diagnosis) had the following reasons for happening: progressive HL (52.5%), complex medical conditions (16.9%), family indecision (9.3%), geographical location (5.9%), and other miscellaneous known (6.8%) and unknown factors (8.5%). In our reality, as a developing country, we believe that socioeconomic factors such as low levels of parental schooling and poor management of referrals from the public network are critical reasons to add to that list.

Despite not having been designed to elucidate the reasons for our children's late implantation, the fact is that geographical distances from specialized centers are a challenge for many patients. Previous surveys within our sample show that more than 50% of our patients live in the countryside regions of our state and need to travel for many hours every time they need the assistance of a physician, audiologist, or speech therapist. In a study conducted by our group (unpublished data), many of those patients had not received proper speech therapy (sessions with a frequency lower than once a week or with professionals not trained for auditory-verbal therapy) in their hometowns.

## Conclusion

The present retrospective study describes the results obtained by a public CI Program, and it has helped to raise awareness about important issues regarding language development in this sample, as aforementioned. We found an elevated age at first implantation, a large group of children with unknown etiology, and a probable association between better language development and earlier age at implantation.

Learning to speak is a complex process, even for children with normal hearing. When HL exists, the complexity is much higher due to factors associated with the patient's medical profile and the social context, which ultimately influences the adherence to therapy required after HL treatment. Nevertheless, many other very important factors remain to be explored, which could help explain the rough results shown. Lack of adherence to speech therapy as well as the widespread effects of auditory deprivation on brain development (which affects the capacity to process information beyond the auditory system) are also crucial for the development of oral language, 14 22 23 24 25 and need to be better examined in our population.

Nonetheless, although all aforementioned reasons are of considerable importance – yet to be classified on a scale of magnitude –, it would be unfair not to mention the concern about our ability to offer the number of consultations needed for proper follow-up. Regardless of age (but especially for children), accurate programming and mapping after CI surgery is a significant contributor to postoperative performance. Frequent appointments are necessary, especially in the first year following activation, to maximize audibility. 26

We hope our findings can serve as a starting point for improvements in assistance based on the data we already have and for future studies that can deepen the knowledge on the subject. Moreover, we expect to see more groups in developing countries willing to publish their results. Hopefully, children in developing countries will achieve better results if all of those objectives are reached.
